# Silk-trehalose seed coating technology preserves *Rhizobium tropici* viability and enhances zinc biofortification in common bean under marginal soil conditions

**DOI:** 10.3389/fpls.2026.1738866

**Published:** 2026-02-19

**Authors:** Manal Mhada, Salma Mouhib, Khaoula Errafii, Adil El Baouchi, Oumaima Zaher, Dachena Romain Gracia, Augustine T. Zvinavashe, Lamfeddal Kouisni, Benedetto Marelli

**Affiliations:** 1 Agrobiosciences (AgBS), College of Agriculture and Environmental Sciences (CAES), Mohammed VI Polytechnic University (UM6P), Benguerir, Morocco; 2 Africa Genome Center (AGC), Mohammed VI Polytechnic University (UM6P), Benguerir, Morocco; 3 Department of Civil and Environmental Engineering, Massachusetts Institute of Technology, Cambridge, MA, United States; 4 African Sustainable Agriculture Research Institute (ASARI), Mohammed VI Polytechnic University (UM6P), Laayoune, Morocco

**Keywords:** biofortification approach, marginal lands, *Rhizobium tropici*, seed coating, trehalose

## Abstract

Sustainable food production requires global access to fertilizers, reducing yield gaps in marginal lands, and decarbonizing the agricultural sector. This study evaluates plant growth-promoting rhizobacteria (PGPRs) preserved in silk-trehalose seed coatings for six months under ambient conditions for their potential to enhance crop yields in challenging soils. Common bean seeds were coated with silk, trehalose, and Rhizobium tropici using a low-tech pan-coating method and tested in greenhouse experiments and field trials across three Moroccan experimental farms with contrasting soil types (favorable, low organic matter, and saline). Rhizosphere microbial communities were characterized using 16S rRNA gene sequencing, and grain nutritional quality was assessed by ICP-OES analysis. Coated seeds showed improved vigor, larger biomass, and enhanced root architecture compared to non-coated seeds under stress conditions. Field trials demonstrated that seed treatment was associated with 50–75% increases in yield parameters and a 53% increase in grain zinc concentration, depending on soil conditions. Additionally, the rhizosphere of treated plants exhibited an enhanced presence of beneficial microbes, such as Bacillus and Acidobacteria, without disrupting native bacterial communities. This low-tech seed coating approach offers a promising sustainable solution for enhancing food production and nutritional quality in resource-limited, environmentally challenged regions.

## 1 Introduction

A projected 70% increase in crop production will be necessary to feed a world population of ~ 9.8B people by 2050 (Food and Agriculture Organization of the United Nations, 2017) while the agrifood system is required to decarbonize food production and address challenges imposed by climate stressors (Schmidt, 2022), plateaued arable land (Ramankutty et al., 2018), decreased soil quality (Montanarella et al., 2016; Qiao et al., 2022), and scarce access to resources such as water (Falkenmark et al., 2019) and fertilizers (Chowdhury et al., 2017). The overlap of regional hotspots for climate change and population growth will exacerbate the strain on local food production (Müller et al., 2021) in regions already facing food security and high-yield gap challenges (Rippke et al., 2016), such as the Maghreb and the Sahel (van Ittersum et al., 2016). In this scenario, enhancing crop production in the African continent is a key objective to guarantee global food security given the projected >1B people population increase by 2050 (+80%) (Gu et al., 2021), a current agricultural yield gap between 30% and 80%, and the forecast increase in temperature (Gao et al., 2025) and drought in North-East, Southern, South-West, and Sahel regions (Serdeczny et al., 2017). Mitigation of extreme weather events will also be particularly challenging for the world’s 500 million smallholder farms that provide livelihoods for more than 2 billion people. In Africa, 70% of the crops are produced by farms <5 ha, with ~80% just reaching subsistence food production with little to no access to fertilizers and high-quality seeds due to a combination of transportation costs, counterfeiting, and lack of infrastructure (Lowder et al., 2016).

**Figure 1:**
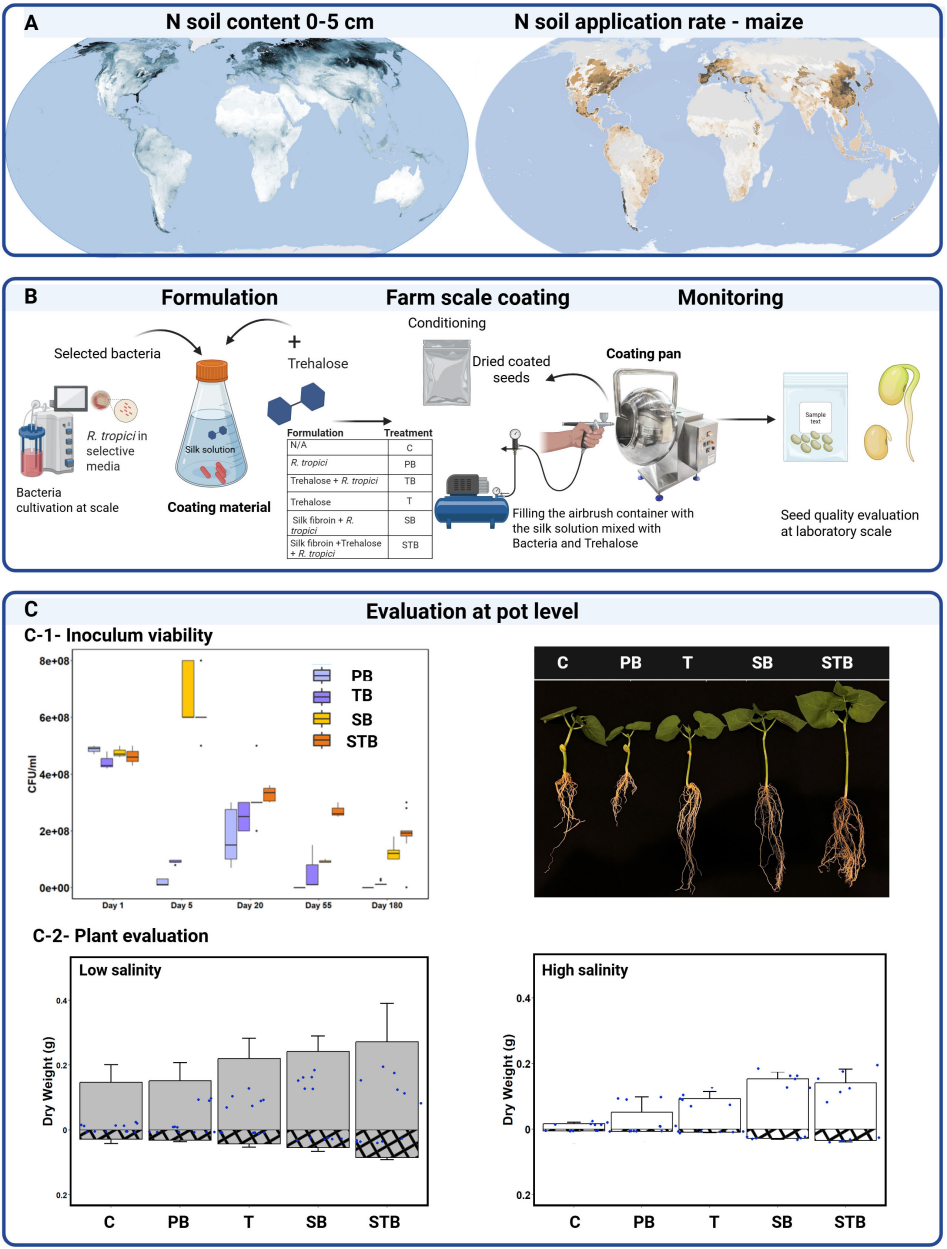
Global nitrogen distribution and microbial seed coating technology for sustainable common bean cultivation under environmental stress. **(A)** Global soil nitrogen distribution and application rates for maize (Poggio et al., 2021). **(B)** Bacterial formulation and seed coating workflow including *R. tropici* cultivation, trehalose coating, and quality control. (C-1) Inoculum viability and visualization of Common bean performance under salinity stress (60 mM) using different treatments and (C-2) plant performance under salinity stress for treatments: control **(C)**, Priming with bacteria (PB), priming with trehalose (T), Coating bacteria with silk (SB), and bacteria with trehalose and silk (STB).

The urge to close the yield gap while mitigating the environmental impact of the agrifood systems has increased the focus on new technologies (Horton et al., 2021) that can achieve ‘sustainable intensification’ in communities with underperforming crop production (Teng et al., 2024) by providing alternatives to mere management through the application of inputs such as synthetic fertilizers (**Figure 1A**) (Poggio et al., 2021). In the case of nitrogen (N) fertilizers, their synthesis consumes 1-3% of the world’s energy, and the associated costs make them scarcely available in rural and emerging areas. The introduction of nitrogen in topsoil is, nonetheless, essential for intensive plant growth, making the quest for new fertilizers increasingly urgent. Plant growth-promoting rhizobacteria (PGPRs) are biofertilizers that can readily fix nitrogen, make phosphorus bioavailable in the soil, and boost plants’ health by conferring adaptive advantages to plants, enhancing their toleration to environmental stressors (Kushwaha et al., 2020; Tian et al., 2024). However, PGPRs adoption, especially in regions where agriculture is predominantly small-scale and less technical, remains low due to the challenges posed by integrating living organisms into the agricultural practices of smallholder farmers and a lack of consistent performance (Raimi et al., 2021). PGPRs do not survive desiccation and are routinely applied at the farm level by priming seeds within hours before sowing (Berninger et al., 2018; Paravar et al., 2023), which requires transportation in liquid form, handling at high concentration, and rapid deployment in the soil (Herrmann and Lesueur, 2013). Encapsulation, preservation, and delivery of PGPRs in a seed coating format can alleviate the technical difficulties associated with managing biofertilizers in a rural environment by enabling long-term storage directly on the seed surface without the need to handle living systems and sow the treated seeds in a timely manner (Malusá et al., 2012). Among the different options that have been explored to formulate seed coatings for PGPRs treatments, silk and trehalose-based solutions bring merits of biodegradation in soil (Pereira et al., 2004; Zvinavashe et al., 2022), preservation of microbes (Mhada et al., 2021; Zvinavashe et al., 2019), and biopolymer design to mitigate abiotic stressors such as water scarcity (Augustine T Zvinavashe et al., 2021). However, previous efforts did not provide evidence of scalability, long-term storage efficacy, and agronomic impact in relevant farm settings. Here, we demonstrate the performance of silk-trehalose seed coating in preserving *Rhizobium tropici* on the surface of common bean seeds (*Phaseolus vulgaris)*, the beneficial effects of the biofertilizer delivery on the rhizosphere composition post-harvest, and the efficacy in increasing yield and quality of crops grown in marginal land under environmental stressors. Seeds were treated using low-tech tools that can be deployed in rural areas and were grown in three different experimental farms in Morocco characterized by sandy, nitrogen-deficient, and saline soil conditions, without the application of nutrients.

## 2 Methods

### 2.1 Material development

#### 2.1.1 Silk solution preparation and evaluation

To ensure the preservation of the same protocol we designed a workflow where silk was prepared based on the protocol described by Rockwood et *al* (Rockwood et al., 2011). and applied for seed coating purposes by Zvinavashe et al., 2019. But instead of using cassettes for the dialysis, we adopted the dialysis membrane to coat seeds for the pots and field experiments.

#### 2.1.2 Inoculum preparation

*R. tropici* CIAT 899 was selected based on its growth-promoting traits under drought stress and its ability to colonize and nodulate on common bean from previous studies (Shamseldin and Velázquez, 2020). Besides, this strain is a non-spore-forming bacterium with limited viability outside the soil and poor survival post-desiccation (Vriezen et al., 2007). *R. tropici* CIAT 899 was obtained from Universidad Nacional Autonoma de Mexico. This strain was selected based on its growth-promoting traits under drought stress and its ability to colonize and nodulate common bean (Shamseldin and Velázquez, 2020).”

To prepare the bacterial suspensions, the strain was streaked on Plate Count Agar (PCA) from glycerol stocks stored at − 80°C and incubated for 72 h at 28°C to facilitate gentle recovery of cryopreserved cells (Prakash et al., 2013). The extended incubation period was required due to the slow growth rate characteristic of *Rhizobium* species (Ondieki et al., 2017; Somasegaran and Hoben, 1994). A single colony from the PCA was transferred to a tube containing 10 mL of yeast extract mannitol broth (YEM), the specific medium for *Rhizobium*. Starter cultures were obtained by incubating the broth for 48 h at 28°C to reach late exponential phase.

The 48-h tube culture was then used to inoculate flasks containing YEM broth for large-scale inoculum preparation. The inoculum for field inoculation was prepared in flasks using YEM broth containing *R. tropici*. YEM media were prepared using a standard composition (0.5 g of yeast extract, 15 g of mannitol, 0.5 g of K2HPO4, 0.2 g of MgSO4.7H2O, and 0.1 g of NaCl per liter, with the pH adjusted to 7.0). Each flask containing broth was inoculated with *R. tropici* and incubated at 28°C for 72 h under shaking (100 rpm) conditions.

Following incubation, bacterial cells were harvested by centrifugation at 6,000 × g for 10 min at 4°C (INFITEK, Centrifuge model CFG-16BY/CFG-16SY). The supernatant was discarded, and the cell pellet was washed twice with sterile physiological saline water (0.85% NaCl) to remove residual medium components and extracellular metabolites. The washed cells were resuspended in sterile physiological water. The optical density (OD600) was measured, and the cell suspension was adjusted by dilution with sterile physiological water to achieve a target concentration of 8–9 log CFU/mL before seed inoculation. Final cell density was verified by serial dilution plating on YEMA (28°C, 48 h).

### 2.2 Seed coating technology

#### 2.2.1 Pan-coating process

Seeds were coated using a low-tech pan-coating method suitable for deployment in resource-limited settings (**Figure 1**). Unlike previous implementations that required laboratory-grade equipment and controlled conditions, our approach utilizes a semi-industrial rotating pan system with regulated airbrush application that can be adapted to rural settings with minimal infrastructure. This represents a significant advance in practical implementation, as it eliminates the need for specialized dialysis cassettes used in earlier studies and reduces the technical barriers to adoption.

Seeds were initially sterilized with 2% bleach solution, rinsed thoroughly with tap water, and dried completely to prevent fungal contamination during subsequent germination testing. The rotating pan was sanitized with 70% ethanol and preheated to 50°C, gradually stabilizing at 45°C during operation. Seeds were placed at the pan’s center, and the silk-trehalose-bacteria suspension was gradually applied via regulated airbrush system while the pan rotated at 40 rpm, maintaining continuous rotation until uniform coating was achieved. Critical parameters were monitored throughout the 10-minute coating process: temperature was maintained at 40°C, and seeds were observed for the development of characteristic hyaline appearance, enhanced surface brightness, and uniform coating texture. Between different seed treatments, thorough cleaning of both the pan and airbrush was mandatory. The process was terminated after the standardized 10-minute rotation period, followed by careful seed collection. Coated seeds were then air-dried at room temperature overnight. Post-coating procedures included immediate cleaning and sanitization of all equipment to ensure readiness for subsequent experiments.

#### 2.2.2 Seed quality assessment and viability measurements

Quality assessment of coated seeds was conducted through multiple analyses: germination potential was evaluated, coating uniformity and surface morphology were characterized using Scanning Electron Microscopy (SEM), and bacterial viability was quantified through selective cultivation on YEM media specific for *R. tropici*.

Germination tests were conducted following a standard laboratory protocol. For each treatment, 10 seeds were placed in Petri dishes lined with moist filter paper. Three replicates were used per treatment. Seeds were incubated in a dark germination chamber at 25°C and 80% relative humidity. Moisture was maintained by adding sterile distilled water as needed throughout the test period. Germination was assessed daily for 15 days, and seeds were considered germinated when the radicle emerged. Final germination percentage was calculated at the end of the test period.

Bacterial viability was quantified through selective cultivation on YEMA specific for *R. tropici*. At each sampling time point, three coated seeds were aseptically transferred to [VOLUME, e.g., 10 mL] of sterile physiological saline (0.85% NaCl) and vortexed for 2 min to dislodge bacterial cells from the seed surface. The resulting suspension was serially diluted (10^−1^ to 10^−8^) in sterile physiological saline. Aliquots from appropriate dilutions were spread-plated in triplicate onto YEMA plates. Plates were incubated at 28°C for 48–72 h, and colonies were counted. Results were expressed as colony forming units per seed (CFU/seed) or per mL of initial suspension (CFU/mL).

We conducted a six-month preservation study of *R. tropici* on common beans, using a formulation made of silk fibroin and trehalose. Coated seeds were stored at room temperature (25°C) under dry conditions in zip sealed aluminum sterile bags. Bacterial load was monitored at regular intervals using the viability assessment protocol described above. The silk fibroin-trehalose formulation effectively preserved bacterial viability, maintaining concentrations at approximately 10^8^ CFU/ml throughout the study period.

### 2.3 Experiments implementation

#### 2.3.1 Greenhouse trial

Greenhouse experiments were conducted at Mohammed VI Polytechnic University (UM6P) experimental facilities. The Temperature at the greenhouse during the experiment was maintained at 25°C (day) and 16°C (night), with relative humidity of 49% and natural photoperiod.

Common bean seeds were sown in plastic pots (20 cm diameter × 25 cm height) containing a mixture of sandy and agricultural soil from Benguerir station (3:1 v/v) to ensure proper drainage.

Prior to the experiment, soil physicochemical properties were analyzed (**Table 1**). Soil pH and electrical conductivity (EC) were measured in a 1:5 soil-water suspension. Organic matter was determined by the Walkley-Black method. Nitrogen forms (NO_3_^−^ and NH_4_^+^) were determined using the Skalar method with KCl 1/5 extraction. Total nitrogen was analyzed according to NF ISO 11261 (1995). Available phosphorus, exchangeable cations (K_2_O, Na_2_O, CaO, MgO), and micronutrients (Fe, Mn, Zn, Cu, B) were determined following NF X 31-108 (2024). Calcium carbonate content was measured by the volumetric method.

**Table 1:** Physicochemical properties of the soil mixture used in greenhouse experiments.

Parameter	Value	Unit
General soil properties		
pH (1:5 H_2_O)	8.4 ± 0.00	–
Electrical conductivity (EC)	1.43 ± 0.05	mS/cm
Organic matter (OM)	2.76 ± 0.05	%
Calcium carbonate (CaCO_3_)	3.27 ± 0.05	%
Nutrient content		
Total nitrogen (N)	0.08 ± 0.00	%
Nitrate (NO_3_^−^)	7.65	mg/kg
Ammonium (NH_4_^+^)	3.60	mg/kg
Available phosphorus (P_2_O_5_)	30.33 ± 0.47	mg/kg
Potassium (K_2_O)	387.67 ± 5.79	mg/kg
Calcium (CaO)	10,704.67 ± 5.44	mg/kg
Magnesium (MgO)	980.67 ± 1.70	mg/kg
Sodium (Na_2_O)	1,589.33 ± 4.92	mg/kg
Iron (Fe)	6.29 ± 0.06	mg/kg
Manganese (Mn)	10.82 ± 0.03	mg/kg
Zinc (Zn)	0.68 ± 0.02	mg/kg
Copper (Cu)	0.88 ± 0.00	mg/kg
Boron (B)	0.82 ± 0.01	mg/kg

Values represent mean ± standard deviation (n = 3). Nitrogen forms were determined using Skalar method with KCl 1/5 extraction. Total nitrogen was analyzed according to NF ISO 11261 (1995). Exchangeable cations and micronutrients were determined following NF X 31-108 (2024).

The soil analysis revealed several characteristics relevant to the objectives of this study. First, the alkaline pH (8.4) and calcareous nature (CaCO_3_: 3.27%) are typical of semi-arid Mediterranean soils, which often present challenges for nutrient availability and plant growth. Second, nitrogen content was low (total N: 0.08%; NO_3_^−^: 7.65 mg/kg; NH_4_^+^: 3.60 mg/kg), creating nitrogen-limiting conditions essential for evaluating the nitrogen-fixing capacity of *R. tropici* inoculation. Third, zinc availability was low (0.68 mg/kg), providing an opportunity to assess the potential of seed coating treatments to enhance zinc biofortification in common bean. Fourth, the electrical conductivity (1.43 mS/cm) indicated low initial salinity, establishing an appropriate baseline for the salinity stress experiments. Available phosphorus (30.33 mg/kg) and potassium (387.67 mg/kg) were at moderate levels, ensuring that these nutrients would not be limiting factors in the experiment.

For salinity stress treatments, pots were irrigated with either tap water (control) or saline solution (60 mM NaCl) maintaining 70% of field capacity throughout the experiment. Saline solution was applied at full concentration (60 mM NaCl) from the onset of irrigation to simulate sudden salinity exposure, which commonly occurs in field conditions following irrigation with saline groundwater.

This salinity level was selected to reflect real-world conditions in Mediterranean and North African agricultural zones, where soil salinity frequently reaches or exceeds this threshold (Chaieb et al., 2019). Additionally, this concentration represents a critical point for common bean productivity in marginal lands (Hiz et al., 2014) while remaining within the range where intervention strategies can be effective (Bourgault et al., 2010; Torche et al., 2018). At the end of the experiment, soil electrical conductivity was measured to confirm salinity levels. Final soil EC values reached 5.6 dS/m in salt-treated pots compared to 1.5 dS/m in control pots, confirming successful salt stress imposition.

The experimental design followed a completely randomized design with six replicates per treatment. Five treatments were evaluated: (1) Control (untreated seeds), (2) Priming with *R. tropici* (B), (3) Priming with Trehalose (T), (4) Coating with Silk and *R. tropici* (SB), and (5) Coating with Silk, *R. tropici*, and Trehalose (TB). Three seeds were initially sown per pot. Ten days after emergence, seedlings were thinned to one plant per pot. The seedling located in the center of the pot was retained, while the lateral seedlings were carefully removed to minimize root disturbance. Plants were monitored for 4 weeks post-emergence, after which they were harvested for biomass and physiological measurements. No additional fertilizers were applied during the experiment to evaluate the direct effects of the treatments under nutrient-limited conditions. This approach allowed assessment of the biofertilizer potential of *R. tropici* inoculation without confounding effects from external nutrient inputs.

#### 2.3.2 Multilocation trials

##### 2.3.2.1 Sites characteristics

Field activities were carried out during the 2020–2021 growing season in three distinct agro-ecological zones in Morocco. The first experiment was conducted at the experimental platform in Laayoune (27°11’01.2”N 13°19’29.4”W), the second at Tassaout, an INRA (National Institute of Agronomic Research) experimental farm (31°49’12.6”N 7°26’16.1”W) and the UM6P experimental farm in Benguerir (32°13’08.3”N 7°53’23.5”W). All experimental sites are characterized by arid conditions, with annual rainfall of approximately 90 mm. Soil samples were taken for preliminary assessment of the nutritional status and salinity level. The soils are classified as sandy-loamy with different gradients and different for other parameters as detailed in **Table 2**.

##### 2.3.2.2 Experimental design

To account for spatial variability, each field trial was arranged in a Randomized Complete Block Design (RCBD) with five replicates and five treatments: (1) Control, (2) Priming with Bacteria, (3) Priming with Trehalose, (4) Coating with Bacteria, and (5) Coating with Bacteria and Trehalose.

A drip irrigation system was employed, with lateral lines spaced at 0.40-m to align with the rows and emitters spaced at 0.15 m to match plant positions within rows. The irrigation sectors were equipped with valves and pressure gauges to maintain an operating pressure of 1 bar and an emitter flow rate of 4 L h−1. As this study was conducted on a demonstration platform, irrigation was applied following local farmer practices to ensure relevance and transferability of results to smallholder farmers in the region.

Prior to sowing, fields were manually prepared following local farmer practices, including weeding, shallow tillage, and bed preparation. Sowing lines were established following common bean (*Phaseolus vulgaris)* cultivation practices. Navy beans (*Phaseolus vulgaris*), also known as Haricot Beans, were used in this study. This variety is widely cultivated in Morocco for both fresh pod consumption and dry seed production, and is valued as an important export crop.

### 2.4 Microbiome evaluation

To reveal the impact of seed coating and priming treatments on soil microbial communities, we used advanced metagenomic analysis techniques. While traditional methods provided limited insights into soil microbiome complexity, the advent of 16S amplicon sequencing has revolutionized our understanding of soil microbial ecology. Our workflow focused on comprehensive microbiome characterization through bacterial 16S rRNA gene sequencing. Despite challenges with soil-derived PCR inhibitors, a robust sampling strategy produced statistically significant datasets for comparative analysis at flowering and harvest stage of the soil communities. This approach enabled us to track temporal shifts in microbial populations and evaluate the ecological impact of different seed treatments on rhizosphere communities.

For microbiome analysis, soil samples were collected from 3 randomly selected plants per treatment per block at two timepoints (flowering and harvest stage), providing 3 replicates per treatment per timepoint (n=3). This sampling strategy ensured sufficient statistical power for detecting community-level changes while accounting for spatial heterogeneity in the field.

#### 2.4.1 DNA extraction

The starting input amount was 250 mg of soil. DNA was extracted using the standard protocol of DNeasy PowerSoil Pro Kit Cat# 47016 (Qiagen) following the manufacturer’s instruction. The final elution volume of extracted was 50 µl in TE buffer. Samples were stored at -20°C until use.

#### 2.4.2 PCR amplification, library preparation and sequencing

The bacterial variable regions V3-V4 of 16S rRNA gene are amplified to build the sequencing library using Fluidigm (Reagent Kit, Access Array™ Barcode Library for Illumina^®^ Sequencers—384, Single Direction, Fluidigm Cat#100-4876) Single Indexing Protocol compatible with Illumina Platform (MiSeq). The Specific primers 515F (5′-CS1-GTGCCAGCMGCCGCGGTAA 3′) and 806R (5′-CS2-GGACTACHVGGGTATCTAAT-3′). Custom Sequence Adapters CS1: ACACTGACGACATGGTTCTACA and CS2: TACGGTAGCAGAGACTTGGTCT were added to each forward and reverse primers. Primers were synthetized and HPLC purified by Alpha DNA (Montreal, QC).

A unique FLuidigm tag was added to each PCR (Polymerase Chain Reaction), and two libraries were constructed for bacteria (one library per 200 samples). Equal amounts of amplified DNA were pooled, followed by a cleaning step with a 0.85 ratio of AMPure XP beads (Beckman Coulter). Libraries were quantified using qubit 4 Qubit dsDNA HS and BR Assay Kits cat # Q32851. Fragment size was determined using Agilent’s Bioanalyzer instrument using Agilent DNA 1000 assay kit Cat # 5067-1504. PCR reactions contained 1 ng of DNA, and KAPA HiFi HotStart Ready Mix. The Ready Mix contains KAPA HiFi HotStart DNA Polymerase (0.5 U per 25 µL reaction) in a proprietary reaction buffer containing dNTPs (0.3 mM of each dNTP at 1X), MgCl2 (2.5 mM at 1X) and stabilizers (Roche). PCR was run using a thermocycler with an initial denaturation of 94°C for 2 minutes, followed by 30 cycles of 94°C for 30 seconds, 55°C for 30 seconds, and 72°C for 30 seconds, with a final elongation at 72°C for 7 minutes. PCR reaction, barcode multiplexing, and Illumina MiSeq were performed at UM6P.

The sequencing has been performed on Miseq instrument using MiSeq Reagent Kit v2 (600-cycles) PE Cat # MS-103-1003.

### 2.5 Data collection and analysis

To evaluate if seed coating can become an agronomically useful management practice, we have developed a diagnostic tool using a transdisciplinary approach that includes material sciences, plant sciences, microbiology, and genomics, going from laboratory set up to greenhouse and field pilot (**Figure 1**).

#### 2.5.1 Data collection protocol

##### 2.5.1.1 Bacterial viability test

To evaluate bacterial viability over time, coated seeds were analyzed for bacterial load at regular intervals over a six-month storage period. Seeds were sampled on 5, 20, and 55 days initially, followed by a final assessment at six months post-coating. For each time point, three coated seeds were randomly selected and suspended in 9 mL of sterile physiological water (0.9% NaCl). The suspension was vortexed for 2 minutes to detach bacteria from the seed surface. Serial dilutions were prepared (10–^1^ to 10^-8^) and 100 µL of appropriate dilutions were plated on YEM (Yeast Extract Mannitol) medium specific for *R. tropici*. Plates were incubated at 28°C for 48–72 hours before counting. Colony Forming Units (CFU) were calculated and expressed as log CFU/mL. The bacterial population was monitored and maintained at an initial concentration of 10^8^ CFU/mL across all treatments. Each assessment was performed in triplicate (n=3).

##### 2.5.1.2 Measurement of plant morphological, physiological and yield traits

*The greenhouse experiment* evaluates plant response to salinity stress using 60 mM NaCl (equal to 6 dS/m) considered as high salinity level for *Phaseolus vulgaris*, with parallel controls maintained under non-saline conditions. Plants were monitored throughout their growth cycle, with biomass accumulation measured through Root and Shoot Dry Weight (RDW and SDW) determinations. For biomass analysis, plant material was carefully separated at the collar region into root and shoot portions from six individuals (n=6). Root systems were gently washed to remove substrate while preserving root architecture and hair development. Biomass samples were oven-dried at 70°C for 48 hours before weighing. Root architecture was evaluated using WinRHIZO™ software.

*For field experiments*, plant morphological and physiological parameters were collected systematically throughout the growing season, where analysis was performed with 5 repetitions using sample number n=12 per treatment. At harvest, plants were carefully extracted from each treatment plot, with roots gently separated to preserve the root system integrity. Following careful washing with tap water, shoots were separated from roots at the collar region for individual analysis. Root systems were subjected to detailed architectural analysis, including length measurements from the collar region by scanning and analyzing using WinRHIZO™ software. For dry mass determination, root samples were oven-dried at 70°C for 48 hours before weighing. Throughout the growing period, plant height, vigor, and physiological activity were monitored. NDVI (Normalized Difference Vegetative Index) was measured using Polypen to assess photosynthetic activity, while seed quality was evaluated through Hundred Seed Weight (HSW) measurements using GrainScan (OPTOAGR PMG 101) with 5 repetitions.

##### 2.5.1.3 Measurement of the mineral concentration

Analysis was conducted on six samples from seeds harvested from each treatment. Mineral concentrations were determined using ICP-OES (Inductively Coupled Plasma-Optical Emission Spectroscopy) analysis following acid digestion. 500 mg of each sample was digested in 6 mL concentrated HNO3 (70%) at 90°C for 60 minutes using a QBlock digestion system (Ontario, Canada). The digestion process was enhanced by adding 3 mL H2O2 (30%) followed by an additional 15-minute heating step at 90°C. After adding 3 mL HCl (6 M), samples were cooled to room temperature, diluted to 10 mL final volume, and filtered. Fe, Ca, and Zn concentrations were measured using inductively coupled plasma-optical emission spectroscopy (ICP-OES; ICAP-7000 Duo, Thermo Fisher Scientific, France). Standard curves were generated using serial dilutions (0.1–10 mg L^−1^) for each element. Method validation was performed using laboratory reference materials and NIST standard references to ensure analytical accuracy and precision.

#### 2.5.2 Bioinformatics analysis

Raw sequencing output files (BCL format) generated by the Illumina MiSeq platform were converted to paired-end FASTQ files and demultiplexed based on sample-specific Fluidigm barcodes using Illumina’s bcl2fastq conversion software (**Supplementary Data S1**). Adapter and primer sequences were removed prior to downstream analysis. Sequence processing and analysis were performed in R using the DADA2 pipeline. Quality profiles of forward and reverse reads were inspected, and low-quality bases were trimmed. Reads containing ambiguous bases or exceeding the expected error thresholds were removed. High-quality reads were then dereplicated, and exact amplicon sequence variants (ASVs) were inferred using the DADA2 error-modeling approach. Paired-end reads were merged based on overlap, and chimeric sequences were identified and removed using the consensus method implemented in DADA2.

Taxonomic assignment of ASVs was carried out using a naïve Bayesian classifier against a curated 16S rRNA reference database SILVA, trained on the V3–V4 region. ASVs that could not be assigned at the kingdom level or were identified as non-bacterial were removed from the dataset. Relative abundance was calculated by normalizing ASV counts to the total number of reads per sample and expressed as percentages. Taxonomic profiles were summarized at different taxonomic levels to characterize bacterial community composition across samples.

All bioinformatics analyses were conducted using visualization of microbial community composition was performed using standard ggplot R package.

#### 2.5.3 Statistical approaches: data analysis

Analysis of variance (ANOVA) was performed on data collected from the randomized complete block design (RCBD) with five replications across environments. Treatment effects were evaluated using a mixed-model approach to account for the nested structure of the experimental design across three locations (Benguerir, Tassaout, and Laayoune). Temporal changes in bacterial populations were analyzed using repeated measures ANOVA to account for time-series measurements during the six-month storage period. Treatment differences were considered significant at p < 0.05, and *post-hoc* comparisons were performed using Tukey’s HSD and Dunnett’s tests. All statistical analyses were conducted using R version 4.1.

For microbial community analysis, diversity indices (Shannon and Simpson’s) were calculated to assess both abundance and evenness of species present in the community. All bioinformatics procedures, including the processing of raw sequencing reads and graphical analysis, were performed using the genomics workbench. Briefly, the Silva Project’s version 138 of SILVA LSU 99% v138.1 for 16S rDNA (Quast et al., 2013) and UNITE (version 8.3) were used to construct the Amplicon Sequence Variants (ASV) table and assign taxonomy to ASV. R and dplyr v2.0.0 were used to determine the relative abundance of taxa.

For biofortification data, standard curves were validated using laboratory reference materials, and measurement uncertainty was calculated following standard protocols.

## 3 Results

### 3.1 Long-term microbial stability and greenhouse studies

Seed-surface biofertilizers can significantly benefit agriculture in the Global South and rural areas where the cold chain is unreliable and handling large volumes of microbes is often not feasible (Bashan et al., 2014). Encapsulation in seed coats reduces the need for farmers to pre-sow soil and apply seed treatments (O’Callaghan, 2016). However, if dried after encapsulation, PGPRs typically suffer from poor viability (upon resuscitation) and high variability in performance, which limits their adoption compared to the high reliability and effectiveness of synthetic fertilizers. In this study, we evaluated the long term (up to six months) *R. tropici* (B) preservation performance of seed coating formulations made by trehalose (T), silk fibroin (S), and their combination (i.e. B, TB, SB, and STB), when sprayed on common beans using farm-relevant applicators such as rotating pans (3 kg of seeds per hour) at an initial bacterial concentration of 10^8^ CFU/ml (**Figures 1A, B**). Upon treatment application, seeds were sealed in air-tight containers and stored in a dry and dark location at a temperature ranging between 20 and 25°C, according to standards provided by the Food and Agriculture Organization of the United Nations (FAO) (FAO, 2014). Seeds treated with STB formulation showed peak bacterial counts between days 5 and 20, followed by a decline and stabilization around day 55. This population dynamics pattern aligns with previous observations of rhizobacteria colonization on seed surfaces (Gao et al., 2022), where initial rapid growth is followed by entrance into a semi-dormant state that preserves long-term viability. At six months post-coating, STB seeds maintained significantly higher bacterial populations compared to the other treatments considered such. B, TB, and SB (**Figure 1**C1), with SB treatment also preserving functional bacterial populations (10^8^ CFU/ml) throughout the six-month period (**Figure 1**C1). This long-term preservation of functional bacterial populations exceeds typical shelf-life requirements for commercial inoculants used in legumes and can be considered sufficient to establish good bacterial inoculation and promote plant growth (Ren et al., 2019). Seeds coated with formulation containing silk (SB and STB) showed a highly significant bacterial charge and limited variability in preservation performance when compared to the other formulations, indicating the key role of the hydrophobic structural protein in providing a reliable formulation to preserve the inoculant during long-term storage (Apiwattanasiri et al., 2022).

In greenhouse studies under low salinity stress conditions (electric conductivity (EC)= 1.1 mS/cm) (**Figure 1C2**), treatments with T, SB and STB significantly (p<0.05) increased root dry weight (RDW) (squared pattern) and shoot dry weight (SDW) (solid pattern), respectively as compared to the negative control (C), untreated seeds and the positive control (agronomy-standard seed priming (PB), using *R. tropici* as inoculant). In plant experiments, TB treatment was not used due to the low performance in the long-term preservation of the inoculant as compared to the standard priming procedure. PB and T treatments showed a significant (p<0.05) increase in RDW and SDW when compared to C. However, the relative performance of the two treatments, one biochemical and the other biological, was not statistically different (p>0.05) for both RDW and SDW. The STB formulation yielded the highest RDW and SDW, increasing biomass by 55.6 ± 12.4% (p<0.05) and by 107.3 ± 25.7% (p<0.05), respectively (**Figure 1**C2) when compared to the negative control, i.e. C, and the positive one. i.e. PB. The presence of trehalose–a known osmoprotectant – in the STB formulation improved the performance of the treatment when compared to SB, with SDW increase by 17 ± 8.5 (p<0.05). In saline conditions (60 mM NaCl – equivalent to EC = 7.1 mS/cm), all treatments showed significant (p<0.05) reduced RDW and SDW when compared to low salinity conditions (**Figure 1**C2). PB and T treatments had statistically significant (p<0.05) but limited effectiveness in mitigating salinity stress, when compared to C. STB treatment resulted in plants with significantly (p<0.05) higher plant biomass (i.e. both RDW and SDW) than C and PB, with SDW increasing by ~300% and ~50%, respectively. When considering the total dry weight (i.e. TDW=SDW+RDW), STB-treated plants had an increase in TDW of ~200% when compared to PB treatment, showing the efficacy of STB in mitigating saline stress, as previously reported (Zvinavashe et al., 2019). No statistically significant difference (p>0.05) was observed between SB and STB, indicating the marginal role of T in the seed coating formulation under high saline stress.

### 3.2 Field studies and microbiome dynamics

**Figure 2:**
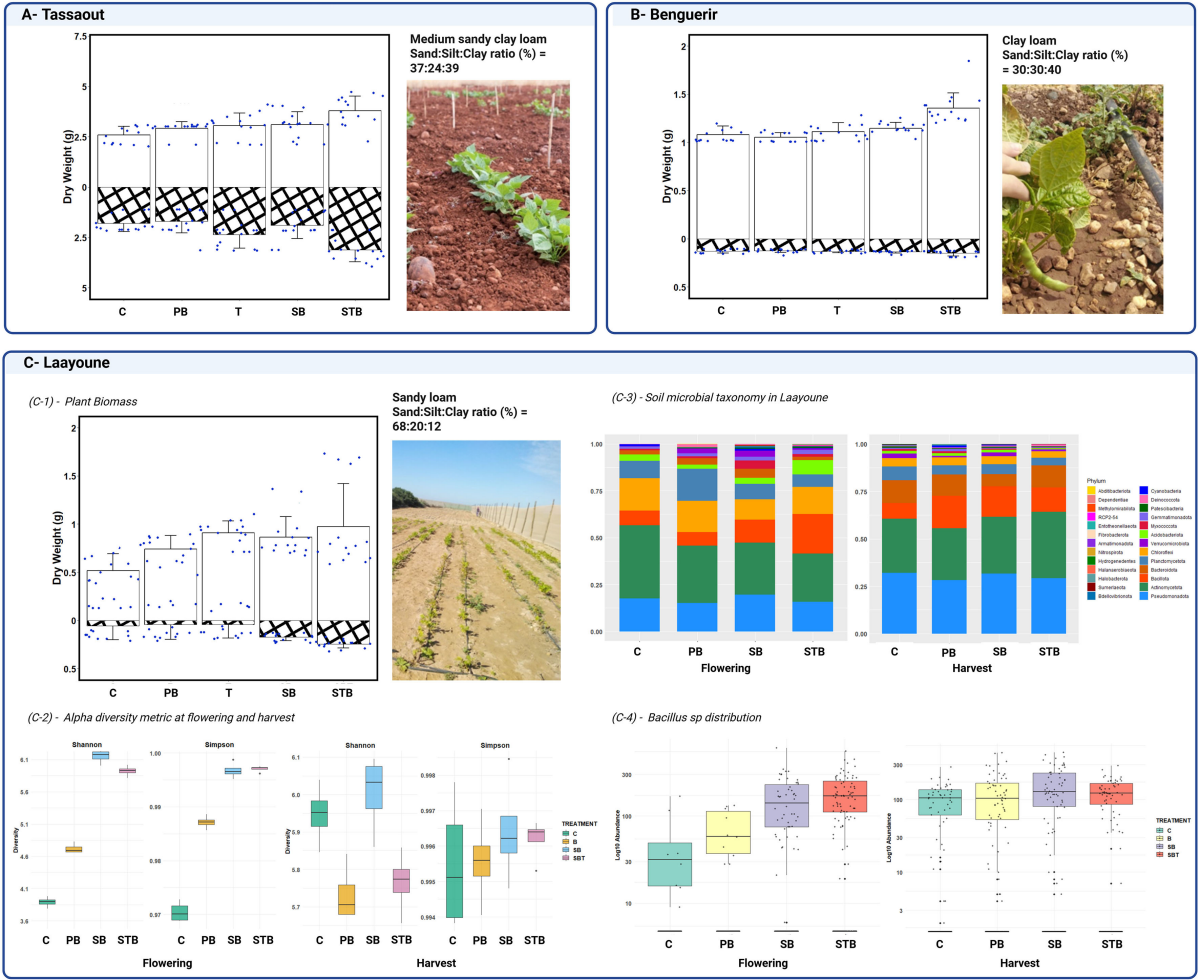
Evaluating plant growth and microbial community shifts in common bean cultivation across diverse soil types. Plant biomass and microbial community analysis at **(A)** Tassaout (Medium sandy clay loam), **(B)** Benguerir (clay loam), and **(C)** Laayoune (sandy loam) sites. (C-1) Plant biomass, (C-2) alpha diversity metrics, (C-3) soil microbial taxonomy, and (C-4) *Bacillus* distribution patterns across treatments.

To support agriculture in rural communities having limited access to fertilizers and healthy soil, the field trials were performed in three experimental farms located in three different zones having different soil types (**Figure 2**). Common beans were sowed in the area of Tassaout (A-Favorable soil conditions), in Benguerir region (B-Low organic matter 0.8%, high soil density), and in Laayoune region (C-High salinity, EC = 4.2 dS/m) – soil analysis for the three locations is reported in the **Table 2**. Seed coating treatments significantly affected (p<0.05) SDW and RDW across the three soil types considered, with STB providing the best performance. In favorable soil conditions, as expected, plants grew better than in the other two soils (i.e. RDW and SDW were higher), with STB-treated plants reaching 39% increase in SDW (3.73 ± 0.67 g) as compared to the untreated seeds (SDW = 2.69 ± 0.37 g). In low organic content soil, STB-treated plants had 21% higher SDW than the C (SDW = 1.33 ± 0.18 g versus SDW = 1.10 ± 0.10 g, p < 0.01) and 21% higher than PB. Highly saline soil severely and negatively affected plant growth. The STB treatment partially mitigated the stress but provided a significant (p < 0.01) growth increase of ~92% (SDW = 0.96 ± 0.46 g) when compared to C (SDW = 0.50 ± 0.18 g). The PB treatment showed a moderate 36% increase in SDW (0.68 ± 0.21 g) compared to the control, but this improvement was substantially lower than that achieved by STB treatment. In addition, T and SB treatments supported better plant growth when compared to the two controls, but their efficacy was significantly (p<0.05) lower than that of STB. Overall, STB treatment showed the most prominent positive effect on plant growth across the three farms considered, with Cohen’s d values of 1.54, 1.92, and 2.07 for Benguerir, Tassaout, and Laayoune region, respectively. This location-dependent variability in treatment effect suggests that the beneficial impact of the STB seed coating technology on plant health is particularly enhanced under challenging soil conditions, where plants typically struggle with multiple stressors.

**Table 2:** Initial soil physical and chemical properties at different experimental sites.

Parameter	Unit	Benguerir (BG)	Tassaout (TST)	Laayoune (LYN)
Site description		Low organic matter high soil density	Favorable soil conditions	High salinity soil
Physical properties				
Textural class	–	Clay loam calcisol	Sandy clay loam	Sandy loam
Sand:Silt:Clay	%	30:30:40	37:24:39	68:20:12
Chemical properties				
pH (1:5 H_2_O)	–	8.1 ± 0.1	8.2 ± 0.2	7.9 ± 0.1
EC	mS cm^−1^	1.1 ± 0.2	0.2 ± 0.1	8.8 ± 0.5

Values represent mean ± standard error (n=3). EC, electrical conductivity.

To assess the efficacy of silk-*R.tropici*-based treatments (i.e. SB, STB) and to enhance the stability of beneficial microbial populations in soils under challenging environments, we investigated the temporal dynamics of the soil microbiome with a focus on highly saline soils with sandy loam texture (**Figure 2**C1).

Analysis of alpha diversity indices revealed distinct patterns in the rhizosphere microbial community composition across treatments and plant developmental stages. The Shannon diversity index showed that SB treatment significantly (p < 0.05) increased microbial diversity at the harvest stage, with values reaching 3.6, representing a 35% increase compared to the values at the flowering stage. In contrast, Shannon diversity in control (C) and priming (PB) treatments remained relatively consistent between the flowering and harvest stages. Simpson’s diversity index further supported these findings, with SB and STB treatments consistently showing higher values (0.95) than control treatments at both plant stages. SB treatment resulted in the most pronounced temporal shift in community structure, suggesting progressive establishment of a more complex microbial community throughout the growing season. PB treatment led to an opposite trend, with diversity metrics decreasing from flowering to harvest, indicating that conventional priming may not sustain microbial diversity throughout the plant lifecycle under saline stress conditions. The elevated diversity indices under silk-based treatments (SB, STB) indicate that these formulations promote conditions that prevent dominance by single taxa, supporting a more balanced distribution of beneficial microorganisms in the rhizosphere, which correlates with the enhanced plant performance observed in field trials. These diversity index values align with those reported in similar studies of rhizosphere communities in saline environments (**Figure 2**C2). For instance, the Shannon diversity value of 3.6 observed in the SB treatment at harvest stage falls within the typical range of 3.2-3.9 reported for halophyte rhizospheres in saline soils (Liu et al., 2022; Mukhtar et al., 2018). Similarly, the Simpson diversity values of 0.95 for both SB and STB treatments are consistent with values (0.92-0.97) observed in other studies of microbial communities in saline environments (Liu et al., 2022; Qiu et al., 2022). The temporal differences in diversity indices between flowering and harvest stages observed in this study reflect similar patterns reported in rhizosphere dynamics under saline stress conditions (Luo et al., 2019). Silk-based treatments showed higher microbial diversity compared to conventional approaches (Shannon index: 3.6 vs 2.8, p<0.05).

Relative abundance analysis revealed that SB and STB significantly (p<0.05) increased microbial diversity during flowering compared to the negative control C and the positive one PB (**Figure 2**C3). C and PB treatments showed substantial presence of Actinomycetota during flowering, while SB and STB resulted in a more balanced community structure and an increased presence of Bdellovibrionota and Methylomirabilota, when compared to the controls. At the harvest stage, microbial communities converged across treatments, although the SB and STB treatments still exhibited unique microbial profiles compared to other treatments. Community composition shifted from flowering to harvest in all treatments, with a reduction in Actinomycetota dominance and an increase in the representation of other phyla.

*Bacilli* abundance was significantly higher in SB and STB treatments compared to C and PB ones (**Figure 2**C4), with no significant (p>0.05) difference between the two silk-based treatments. *Bacillota* emergence and sustained *Methylomirabilota* presence were observed in harvest stage samples, with Bacilli consistently more abundant in STB and SB treatments. Total microbial abundance remained similar across all treatments. STB treatment significantly (p<0.05) increased *Acidobacteria* phyla when compared to both control and PB treatments at harvest stage, with approximately 25% higher relative abundance. This enhancement of *Acidobacteria*, known for their roles in carbon cycling and plant growth promotion under stress conditions, suggests that silk-trehalose-based coatings create favorable conditions for beneficial bacterial communities. Both pot and field experiments showed enhanced secondary root development in treated plants and increased root surface area where beneficial microbes, including *R. tropici*, were maintained.

### 3.3 Plant growth, stress response, and crop yield and quality

**Figure 3:**
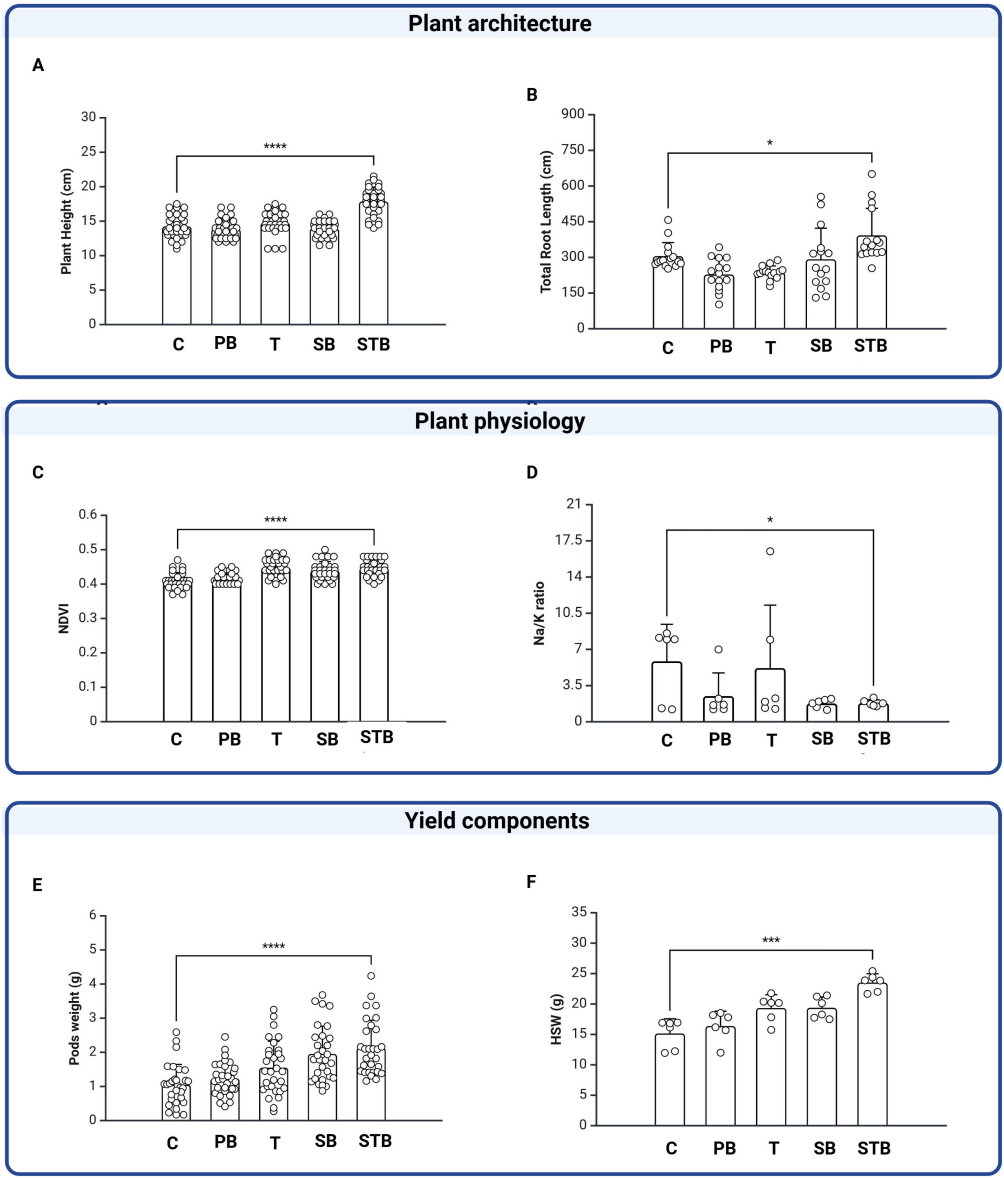
Microbial seed coating improves common bean growth parameters, physiological traits, and yield components. Comparative analysis showing improvements in **(A, B)** plant architecture (height and root length), **(C, D)** physiological traits (NDVI and Na/K ratio), and **(E, F)** yield components (pod weight and hundred seed weight). Statistical significance: *p<0.05, ***p<0.001, ****p<0.0001.

**Figure 4:**
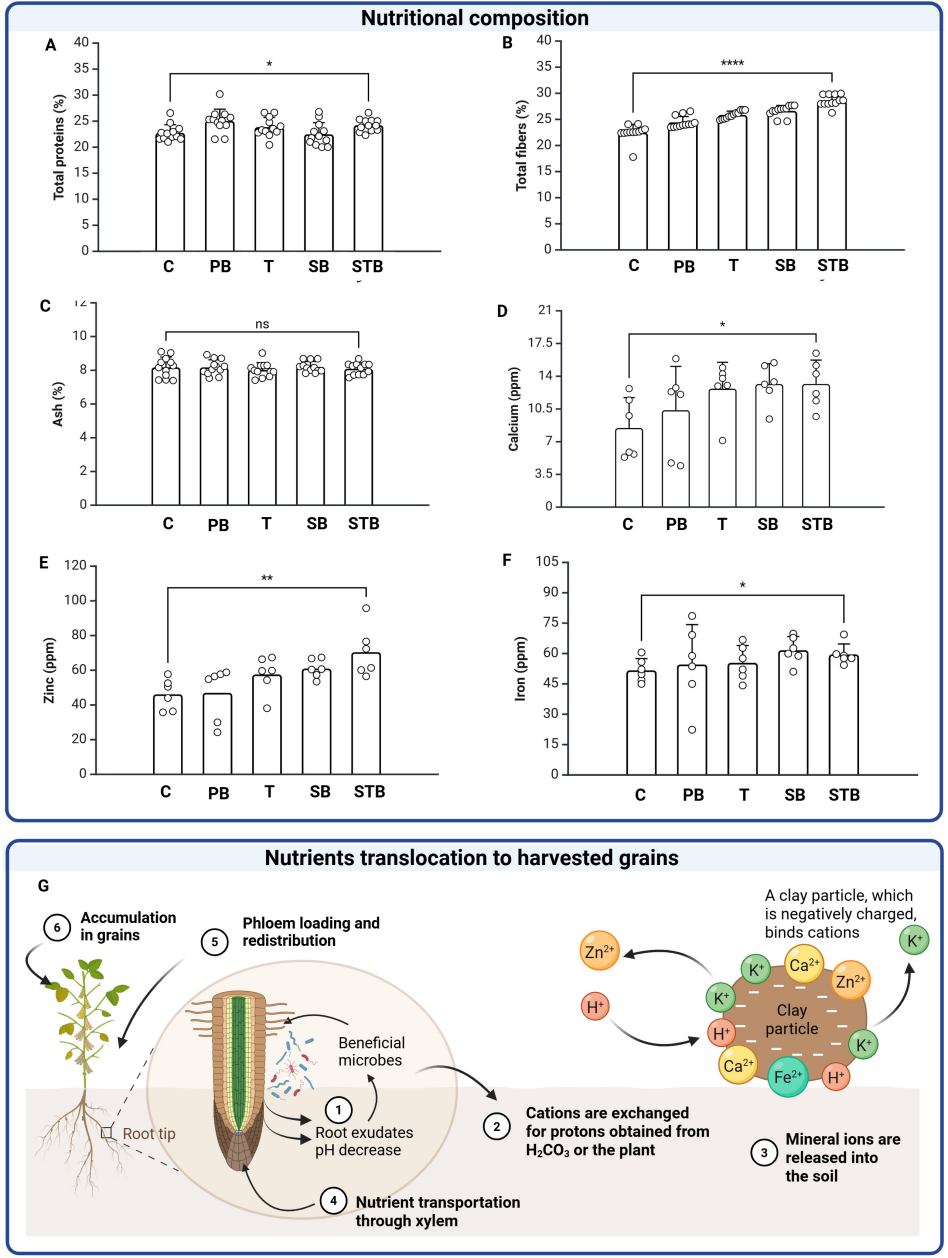
Enhanced nutrient acquisition and translocation in common bean through microbial seed coating. **(A)** Protein content (%), **(B)** Fiber content (%), **(C)** Ash content (%), **(D)** Calcium concentration (ppm), **(E)** Zinc concentration (ppm), **(F)** Iron concentration (ppm). Nutritional quality of harvested grains showing increased calcium, protein, fiber, zinc, and iron. **(G)** Conceptual model of nutrient translocation pathway from soil to grain via root-microbe interactions and vascular transport. Statistical significance: *p<0.05, **p<0.01, ****p<0.0001.

The efficacy of SB and STB seed coatings on plant architecture, physiology, and crop yield were evaluated in the field and compared to C, T, and PB treatments (**Figure 3**). Seed coating with STB resulted in plants with increased plant height (PH, ~25% increase compared to controls, p<0.05) (**Figure 3A**) and enhanced total root length (TRL, ~37% longer than controls, p<0.05) (**Figure 3B**). On the other hand, SB treatment did not significantly affect PH and TRL when compared to controls (p>0.05), indicating a key role played by trehalose when used in combination with silk and the inoculant to mitigate saline stress. T, SB, and STB treatments increased the normalized difference vegetation index (NDVI), which is widely used to quantify the health and density of vegetation. NDVI in STB, SB, and T treated plants were statistically similar (p>0.05) and ~11% higher than C and PB controls (**Figure 3C**, p<0.05). Na/K ratio is a stress parameter commonly used for plants grown in saline soil stress, as an increase in Na^+^ and a decrease in K^+^ uptake (i.e. Na/K imbalance) can disrupt plant development and metabolism. One-way ANOVA test indicated that seed treatments had a significant effect on Na/K ratios (**Figure 3D**), which were highest in plants germinated from C (6.1) and T (5.2) treated seeds. Inoculation with *R. tropici* partially mitigated salt stress, as Na/K ratio decreased for PB (2.3), SB (1.7), and STB (1.5) treatments, with STB showing a ~65% and ~7% reduction when compared to C (p<0.05) and PB (p<0.05). The most striking agronomic outcome was the 105% increase in fresh pod weight in STB seed treatment significantly increased crop yield, with fresh pod weight increasing by ~105% (p<0.05), 72% (p<0.05), 43% (p<0.05), and 16%, (p<0.05) when compared to C, PB, T and SB treatments, respectively (**Figure 3E**). PB, SB and SBT treatments enhanced pulses (i.e. next generation of common bean seeds) quality. Hundred-seed weight (HSW) is a standard metric used in agronomy, plant breeding, and seed technology to assess seed size and density. HSW was significantly (p<0.05) higher in STB-treated plants (23.5 g), when compared to all the other treatments, with a ~57% and ~20% increase when compared to C (13.3 g) and PB (16.5 g), respectively (**Figure 3F**). SB treatment showed a 30% increase in HSW (19.5 g) over Control. Surprisingly, seed treatments positively impacted the nutritional composition of the pulses (**Figure 4**). Proximate analysis showed that protein content was statistically significant (p<0.05) but marginal from a nutritional perspective, increasing from 22 ± 1.5% for C to 25 ± 2.2% for PB and 25 ± 1.2% for STB (**Figure 4A**). Seed treatments significantly affected the fiber percentage in the harvested grains (**Figure 4B**), with values increasing from 22 ± 1.6% for C, to 25 ± 1.3% for PB and 30 ± 1.1% for STB. Ash content was not significantly different (p>0.05) among treatments (**Figure 4C**).

Mineral analysis revealed significant effects on both macro- and microminerals. Calcium concentration, a macromineral essential for human nutrition, was enhanced (13.18 mg/kg) in seeds harvested from STB-treated plants, indicating a ~55% and ~27% increase when compared to seeds harvested from C (8.46 mg/kg) and PB (10.36 mg/kg) plants, respectively (**Figure 4D**). Most notably, grain zinc concentration showed a substantial 53% increase in STB-treated plants (56.5-95.8 mg/kg) compared to C and PB controls (~46 mg/kg) (p<0.05; **Figure 4E**). This increase in zinc concentration, combined with the 105% yield improvement, indicates significant enhancement in total zinc accumulation with important implications for biofortification. Iron concentration showed no statistically significant differences across treatments (**Figure 4F**).

## 4 Discussion

Our study demonstrates that silk-trehalose coatings effectively preserve *R. tropici* viability during long-term storage, addressing a critical barrier to the adoption of biofertilizer adoption in resource-limited settings. Silk-based seed coatings preserved significantly higher bacterial populations than the current standard in agronomy, i.e. priming, with the structural protein creating a protective microenvironment that shields bacterial cells from desiccation while allowing for sufficient gas exchange. The addition of trehalose can enhance the stabilization of cellular membranes during drying (Marelli et al., 2016; Timmusk et al., 2017). This capability may extend the reach of the silk-trehalose formulation beyond agriculture to gene banks to protect indigenous seed-associated microorganisms during transportation and storage (Hill et al., 2021). The demonstrated 6-month, shelf-stable preservation of the inoculant in principle eliminates the need for cold chain and specialized handling procedures while maintaining efficacy, potentially increasing adoption rates in regions where access to synthetic fertilizers is limited by cost and infrastructure constraints.

The comparative results from greenhouse and field studies show that STB coated seeds were associated with improved plant performance across varying environmental conditions, particularly under salinity stress. However, without ST (Silk-Trehalose) controls, the specific contribution of bacterial activity to these improvements cannot be definitively established. *R. tropici* are applied to soil through a single dose via seed coating in known quantities, with topographical control over their delivery, thereby reducing handling and multiple spray efforts. The observed associations between STB treatment and enhanced root architecture and physiological responses suggest potential value for sustainable agriculture in marginal lands. Our findings regarding Na/K ratios and Ca²^+^ uptake provide insights into possible mechanisms of salinity mitigation. The significant reduction in Na/K ratios indicates improved ionic homeostasis, a critical factor in salt tolerance that aligns with previous reports on beneficial bacteria modulating ion transport mechanisms under salt stress. In salt-affected conditions, high sodium levels disrupt cellular processes and nutrient uptake, making our treatments’ ability to lower the Na/K ratio critical for enhancing plant health.

WinRHIZO™ analysis revealed that STB treatment was associated with a 37% increase in total root length (p<0.05; **Figure 3B**) and enhanced secondary root development compared to controls. These root architectural changes may contribute to improved nutrient and water absorption under saline stress, though the specific roles of bacterial activity versus coating material effects in inducing these changes remain to be determined. Previous studies have linked similar root modifications to improved stress tolerance in various crop species (Liu et al., 2007). Our results demonstrate that these adaptations can be induced with a single treatment through the seed coating technology without genetic modification, using wild-type rhizobacteria and micrograms of commodity, generally recognized as safe (US FDA GRAS status) biopolymers applied as seed coating (Marelli and Behrens, 2023), retrofitting existing agricultural tools. At the molecular level, root hair initiation is coupled with localized increases of xyloglucan endo-transglycosylase action, with various XTH isoforms involved in root development (Campobenedetto et al., 2021). The improved NDVI values in STB-treated plants indicate enhanced photosynthetic efficiency, suggesting systemic improvement in plant function under stress conditions. Despite challenges from halophilic bacteria (Ruppel et al., 2013) that can create competitive environments and delay the establishment of beneficial bacteria (Meena et al., 2023). Importantly, nodulation was observed exclusively in bacteria-containing treatments (PB, SB, STB), providing the strongest evidence for bacterial contribution to the observed effects. Symbiotic nitrogen fixation through nodulation cannot be attributed to coating materials alone, confirming successful *R. tropici* establishment and activity in treated plants.

The higher microbial diversity observed in silk-based treatments (SB, STB) during flowering aligns with findings by Jansson et al. who showed that structured delivery matrices enhance early microbial colonization in stress-prone environments (Jansson et al., 2023). SB and STB likely create protected microenvironments that favor their establishment in the rhizosphere upon sowing, shield them from stressors while allowing root interactions, similar to mechanisms previously reported with osmoprotectants (Iordachescu and Imai, 2008). The maintained diversity in silk-based treatments supports theoretical frameworks that link initial colonization patterns to long-term community stability (Kong et al., 2025). The increased presence of *Bdellovibrionota* and *Methylomirabilota* in SB and STB treatments indicates microbial groups associated with enhanced nutrient cycling and plant growth promotion (Dutta et al., 2023), and the emergence of *Bacillota* and sustained *Methylomirabilota* presence in harvest stage samples (Akhtar et al., 2020) suggest that the seed coating technology promotes beneficial succession dynamics under stress conditions. As plants progressed from flowering to harvest, we observed a community shift characterized by reduced dominance of *Actinomycetota* and increased representation of other phyla. This succession pattern resembles that described by Zhao et al. (2023) as being driven by root exudates (Zhao et al., 2023), but the silk-based treatments appear to better facilitate this transition while maintaining key beneficial groups. While silk-*R.tropici*-based treatments exhibited a more balanced community structure, they did not significantly alter the overall microbial abundance, suggesting a non-disruptive approach to rhizosphere engineering essential for sustainable agriculture. This supports functional redundancy in microbial communities, where specific taxa changes don’t disrupt ecosystem functions (Jia and Whalen, 2020). We also observed that SB and STB treatments were associated with increased native microbiota, practically *Acidobacteria* populations known to modulate biogeochemical cycles. Secondary roots can also provide additional carbon sources and greater surface area for beneficial microbe colonization, including the inoculated *R. tropici*. The use of silk and trehalose in seed coating formulation can then enhance microbial stability in challenging environments, where access to carbon sources is restricted (Arellano-Caicedo et al., 2024), by providing rapidly available organic matter in low-carbon soils (Singh and Kumar, 2024), hence facilitating the establishment of plant-beneficial microbial communities.

The enhanced physiological performance and improved plant architecture conferred by STB coating translated into significant improvements in economically important yield parameters through enhanced photosynthetic efficiency, nutrient uptake, and translocation of photo-assimilates to reproductive organs. Fresh pod weight showed a significant increase in STB-treated plants compared to all other treatments (**Figure 3E**), aligning with findings on cowpea (Rocha et al., 2020) and chickpea (Ullah et al., 2020), where enhanced root architecture directly correlates with pod development. HSW was significantly higher in STB-treated plants than other treatments, including PB and T. While this suggests a combined effect of the coating formulation, the individual contributions of silk, trehalose, and R. tropici to yield improvements cannot be distinguished without ST controls. The improvements may result from improved source-sink relationships and enhanced grain filling processes (Ahmad et al., 2021). Beyond yield parameters, the seed coating demonstrated remarkable effects on nutritional composition. STB-treated plants showed significant enhancement in fiber content (+27%), which is particularly valuable for arid regions where livestock feeding relies heavily on legume residues. This increase is supported by findings that certain PGPR strains can modulate carbon allocation and cell wall component synthesis in developing seeds (Bigatton et al., 2024), sharing similarities with gibberellic acid priming effects on oil content in other species (Khan et al., 2020). Most notably for biofortification, grain zinc concentration increased by 53% in STB-treated plants (50–90 ppm), addressing a critical nutritional gap in regions with dietary zinc deficiency (Lowe and Gupta, 2024; White and Broadley, 2009), which has a steep toll on human health, affecting approximately 15% of the global population with higher prevalence in Africa and Asia (Gupta et al., 2020). Zinc deficiency contributes to compromised immunity, impaired cognitive development, and growth stunting. Current interventions like supplementation programs and conventional breeding for biofortification face implementation challenges in resource-limited settings. The STB seed coating approach offers an accessible, rapidly deployable solution associated with simultaneously enhanced crop productivity and micronutrient content without requiring specialized infrastructure. While nodulation confirms bacterial establishment, the relative contributions of *R. tropici* versus coating materials to yield and biofortification improvements require further investigation. Calcium content was also significantly increased in the harvested pulses, which is noteworthy as legumes are not typically an important calcium source in the diet, due to restricted translocation to seeds (Moraghan et al., 2006). This fortification further demonstrates that the seed coating treatment provides nutritional enhancement, offering a viable alternative to resource-intensive methods like genetic transformation or conventional breeding, which often remain inaccessible to smallholder farmers. While previous research explored engineered nanomaterials and delivery of PGPBs via seed priming (De La Torre-Roche et al., 2020) or leaf spraying (Carmona et al., 2024) to biofortify grains, the enhanced pulse nutritional content imparted by a single-dose PGPBs delivery in a seed coating format remained underexplored, adding to the importance of our findings to underpin an accessible technology with beneficial effects on soil and crop quality.

Different soil types significantly influenced the impact of the seed coating treatments on plant growth parameters, with more beneficial relative improvements achieved under challenging soil conditions (particularly high salinity), suggesting that STB and SB address physiological limitations that become more pronounced under stress. The most striking effects of SB and STB coatings were on the physiology of plants grown in highly saline soil. Lower Na/K ratios in STB-treated plants (**Figure 3D**) indicate that the seed coating results in an effective ion homeostasis under saline conditions, explaining the pronounced yield improvements and micronutrient accumulation at the Laayoune farm. In low-carbon, high-soil-density soil, root development is restricted. Nonetheless, STB treatment improved root architecture (21% increase in root biomass when compared to C), enhancing nutrient acquisition despite limited root exploration. In more favorable soil conditions, absolute biomass gains were the largest, but the relative advantage was less pronounced, suggesting that in healthy soil conditions (Xu et al., 2025), SB and STB treatments are less impactful than in marginal land, yet relevant to close the yield gap, one of the major goal for agriculture to support a projected a 9.8 billion world population while limiting carbon emissions and decrease energy use (Frank et al., 2024).

While our findings demonstrate the potential of silk-trehalose seed coatings for delivering beneficial rhizobacteria, a significant limitation of this study is the absence of a silk-trehalose without bacteria (ST). This omission prevents definitive separation of effects attributable to the coating materials from those specifically due to preserved *R. tropici*. Both silk fibroin and trehalose possess known beneficial properties independent of bacterial delivery: trehalose functions as an osmoprotectant and potential carbon source for native soil microbiota (Crowe et al., 1984; Elbein et al., 2003), while silk fibroin provides physical seed protection and releases amino acids upon biodegradation (Rockwood et al., 2011; Zvinavashe et al., 2022). Consequently, the observed improvements in plant growth, yield, and stress tolerance in STB-treated plants cannot be exclusively attributed to bacterial activity, and mechanistic interpretations presented in this study should be considered preliminary.

Nevertheless, indirect evidence supports a significant bacterial contribution to the observed effect. Most importantly, nodulation was observed exclusively in bacteria-containing treatments (PB, SB, STB), providing direct functional evidence of *R. tropici* establishment and symbiotic nitrogen fixation, a process that cannot be attributed to coating materials. Nodulation was assessed in greenhouse experiments; field nodulation data were not collected due to nodule loss during plant extraction from soils. Additionally, 16S rRNA sequencing demonstrated shifts in rhizosphere bacterial communities specifically in inoculated treatments. Furthermore, our previous work characterized silk fibroin effects independently, demonstrating that silk provides physical protection but does not significantly enhance plant growth without beneficial microorganisms (Zvinavashe et al., 2019). However, without ST controls, the relative contributions of coating materials versus bacterial activity to growth promotion and yield improvements remain unquantified. Future studies must include ST controls to resolve this critical question to fully quantify the individual contributions of each component.

Overall, our results demonstrate the efficacy of silk-trehalose coatings in preserving metabolically active, non-spore-forming rhizobacteria in ambient conditions. While previous studies showed preservation of model organisms in controlled environments, our work demonstrates that *R. tropici*, with typically poor desiccation tolerance (Berninger et al., 2018), maintains functional populations (10^8^ CFU/ml) for six months in field-relevant conditions. This addresses what Herrmann and Lesueur identified as the primary barrier to biofertilizer adoption in resource-limited settings (Herrmann and Lesueur, 2013), while our demonstration across multiple agro-ecological zones extends the application domain of silk-based preservation systems beyond their previous investigation in a greenhouse setting (Cassán and Diaz-Zorita, 2016; Augustine T. Zvinavashe et al., 2021). The SB and STB seed coating technologies provide multiple merits in reducing agricultural energy consumption and environmental impact, while increasing yield and food quality. Without additional input, the seed coating significantly provided yield improvements (50-75%), potentially increasing farmer prosperity while reducing dependence on synthetic fertilizers that are difficult to access. The rhizosphere analysis showed enhanced beneficial microbial activity without disrupting native bacterial communities, with an overall improvement in biodiversity indices. Technology can also be easily adopted, giving the ease of handling and minimal infrastructure required to handle and deploy. Additionally, the significant improvements in pulses’ micronutrient content (particularly the 53% increase in zinc content) directly address nutritional security challenges that often accompany food security initiatives. Finally, the six-month PGPBs preservation capability provides practical and industry-relevant performance that aligns with supply chain needs in regions with limited infrastructure. These multidimensional benefits provide a framework for development and adoption of PGPBs delivery via seed coating in marginal lands and resource-limited settings.

## 5 Conclusion

This study demonstrates that silk-trehalose seed coatings effectively preserve R. tropici viability (~10^8^ CFU/seed) for six months under ambient storage conditions and significantly improve common bean performance across diverse marginal soils. Specifically, STB-treated plants showed a 105% increase in fresh pod yield under saline conditions (p<0.05), a 53% increase in grain zinc concentration addressing dietary deficiency concerns, enhanced rhizosphere microbial diversity without disrupting native communities, and improved stress tolerance as evidenced by a 65% reduction in Na/K ratio. However, some limitations should be acknowledged: the absence of a silk-trehalose-without-bacteria control prevents complete attribution of effects to bacterial activity alone, and long-term effects on soil microbiome across multiple growing seasons remain to be evaluated. Nevertheless, this low-tech seed coating approach offers an accessible solution for delivering biofertilizers in resource-limited settings, simultaneously enhancing yield and nutritional quality without requiring specialized infrastructure.

## Data Availability

The data presented in the study are deposited in the NCBI Sequence Read Archive (SRA) repository, under accession numbers SAMN15918186–SAMN15918247.

## Supplementary material

Supplementary Data Sheet 1 Raw 16S rRNA gene sequencing data deposited in Sequence Read Archive (SRA) [SUB15918186] under accession number [SAMN15918186 - SAMN15918247].
